# Oral Fungal Infections: Past, Present, and Future

**DOI:** 10.3389/froh.2022.838639

**Published:** 2022-02-03

**Authors:** Richard D. Cannon

**Affiliations:** Department of Oral Sciences, Sir John Walsh Research Institute, University of Otago, Dunedin, New Zealand

**Keywords:** candidiasis, microbiome, mycobiome, artificial intelligence, machine learning, mucormycosis, COVID-19, SARS-CoV-2

## Abstract

Oral fungal infections have afflicted humans for millennia. Hippocrates (*ca*. 460-370 BCE) described two cases of oral aphthae associated with severe underlying diseases that could well have been oral candidiasis. While oral infections caused by other fungi such as cryptococcosis, aspergillosis, mucormycosis, histoplasmosis, blastomycosis, and coccidioidomycosis occur infrequently, oral candidiasis came to the fore during the AIDS epidemic as a sentinel opportunistic infection signaling the transition from HIV infection to AIDS. The incidence of candidiasis in immunocompromised AIDS patients highlighted the importance of host defenses in preventing oral fungal infections. A greater understanding of the nuances of human immune systems has revealed that mucosal immunity in the mouth delivers a unique response to fungal pathogens. Oral fungal infection does not depend solely on the fungus and the host, however, and attention has now focussed on interactions with other members of the oral microbiome. It is evident that there is inter-kingdom signaling that affects microbial pathogenicity. The last decade has seen significant advances in the rapid qualitative and quantitative analysis of oral microbiomes and in the simultaneous quantification of immune cells and cytokines. The time is ripe for the application of machine learning and artificial intelligence to integrate more refined analyses of oral microbiome composition (including fungi, bacteria, archaea, protozoa and viruses—including SARS-CoV-2 that causes COVID-19). This analysis should incorporate the quantification of immune cells, cytokines, and microbial cell signaling molecules with signs of oral fungal infections in order to better diagnose and predict susceptibility to oral fungal disease.

## Introduction

The oral cavity is a unique ecological niche for microbial colonization. It is an entry portal for the human body through which air, solids, and liquids pass. It provides a variety of surfaces for colonization ranging from the hard non-shedding surfaces of teeth to desquamating keratinized and non-keratinized epithelia. Some individuals have dental appliances which introduce acrylic, polyurethane, ceramic and metal alloy surfaces, that are also colonized. The surfaces in the mouth are kept warm and moist by the constant flow of saliva across them. It is not surprising, therefore, that the human oral cavity supports a complex and dynamic microbiota [1–3]. In general, this microbiota is non-pathogenic and may indeed prevent colonization by overtly pathogenic microorganisms. If the balance of the microorganisms is perturbed by microbial dysbiosis, however, disease and tissue damage and can occur—caused by bacteria, archaea, fungi, or viruses.

Fungi are a minor component of the oral microbiota [4], but can nevertheless cause considerable morbidity [5]. The main cause of oral fungal infections are *Candida* species, less common fungal infections with oral manifestations include mucormycosis, aspergillosis, blastomycosis, histoplasmosis, cryptococcosis, and coccidioidomycosis [5]. To treat oral infections effectively it is important to understand their cause. While visual inspection of disease signs may give an indication of microbial etiology, correct treatment requires knowledge of what is causing the disease, and how. Methods for analyzing microorganisms present in the mouth have evolved rapidly over recent decades with the switch from culturing to DNA detection and sequencing [6].

Having identified possible culprits for oral disease, researchers have tried to ascribe pathogenesis to virulence factors. For example, the tissue degrading enzymes of *Candida albicans* [7, 8] or its cytotoxic molecules [9] have been well-studied. It became apparent, however, that pathogenesis of oral fungal infections depends on host defenses as well as fungal properties [10]. Furthermore, disease may not be caused by a single fungal strain, but may involve the concerted action of several species responding to inter-species communication and the host environment [11].

With continuing changes in the causes of immune compromise and increasing prevalence of co-morbidities there is a need to be able to detect, treat, and preferably prevent oral fungal diseases. This mini-review will chart progress in understanding the etiology of oral fungal diseases, comment on the current state of research, and identify areas for future study.

## Oral Fungal Infections—Past: Disease Etiology, Host Colonization and Fungal Virulence

Fungi are likely to have colonized humans, and caused oral infections, for millennia. Frank Odds, in his monograph on *Candida* and candidosis [12], notes that Hippocrates (*ca*. 460-370 BCE) reports two cases of oral aphthae in his medical work *Epidemics* that could well have been oral candidiasis. It was not until 1839, however, that the fungus causing candidiasis was described by Langenbeck [12]. While other fungi cause oral infections rarely, oral candidiasis came to the fore during the AIDS epidemic of the 1980s as a sentinel opportunistic infection signaling the transition from HIV infection to AIDS [13, 14]. Oral candidiasis has a range of presentations from creamy white plaques to red inflamed mucosae [15] and diagnosis is supported by culturing on selective agar. Presumptive identification can be achieved using selective chromogenic agar such as CHROMagar *Candida* [16], with more definitive identification from rDNA [internal transcribed spacer (ITS) [17]] sequencing or matrix-assisted laser desorption ionization-time of flight (MALDI-TOF) mass spectrometry analysis which is used in clinical diagnostic laboratories [18].

The ability to identify fungi associated with oral lesions led to studies of fungal colonization and virulence, primarily focussed on *C. albicans* and other *Candida* species. Adherence is the first step in colonization [19] and the adherence of *C. albicans* cells to a variety of substrates, including buccal cells [20] and dental acrylic [21], has been investigated. Early studies investigated the adherence of yeast cells to surfaces *in vitro* in the presence of a buffer. Later refinements investigated the role of saliva in adherence to epithelial cells [22], hydroxyapatite [23], and to oral bacteria [24] to relate assays more closely to the *in vivo* situation. There has also been considerable research into how fungi might cause oral disease, and for *Candida* species this has involved studying virulence factors such as secreted aspartyl proteinases [7, 25], phospholipases [26], hyphal formation [27], and the toxin candidalysin [9].

The investigation of the etiology of oral microbial disease has evolved from a reductionist approach, applying the 19th century germ theory of disease in order to identify individual microorganisms responsible for oral diseases, to a holistic approach of examining all the interactions that occur in the oral cavity.

## Oral Fungal Infections—Present: Microbiome Complexity and Immune Interactions

Rapid advances have been made in the last two decades in the analysis of microorganisms inhabiting humans and causing disease, including oral disease. This has been stimulated by the National Institutes of Health-initiated human microbiome project (2007–2016) [6]. Early studies analyzed oral microbiota using DNA-DNA checkerboard hybridization [28]. This technique has the limitation of measuring the abundance of only about 40 microorganisms per sample, and although mainly focussed on oral bacteria, some studies investigated the presence of fungi [29, 30]. The advent of high-throughput DNA sequencing technologies has enabled the analysis of entire microbiomes from oral samples. DNA is extracted from the samples and either rDNA is amplified and sequenced or the DNA is fragmented and it is sequenced in its entirety (metagenomic whole genome sequencing). Amplification and sequencing of bacterial 16S rRNA genes has enabled the identification of hundreds of bacterial taxa from oral samples [31]. Such analyses, however, only detect bacteria, fungal analysis requires the amplification and sequencing of the internal transcribed spacer (ITS) between fungal rRNA genes [17]. This has allowed the description of fungi in oral samples, termed the “mycobiome” [32, 33]. Such studies have reported the fungi present in healthy individuals [32] and those associated with diseases such as caries [34] and periodontitis [35]. DNA sequencing of panels of housekeeping genes enables fungi to be identified to the level of strain types which can be used to study the epidemiology of strain distribution and microevolution of fungal strains within a host [36, 37].

The sensitivity of the DNA sequencing methods has revealed the diversity of microorganisms, including fungi, present in the oral cavity, however this approach is not without its limitations. It generates a massive amount of data that have to be filtered for accuracy and are challenging to analyse. The sensitivity, and low levels of detection of some fungi, raises the question of whether certain fungi detected are transients—present in food, drink or air and are simply passing through the oral cavity—or actual commensals or pathogens [38, 39]. Also, the representation of the mycobiome can be influenced by the ability of the methods used to release DNA from different fungi [38]. Bearing these limitations in mind, mycobiome analyses have confirmed earlier studies using culture methods that the *Candida* genus predominated in oral samples [32–35, 38]. Interestingly however, other fungal genera are also commonly found including *Aspergillus, Malassezia, Cladosporium, Aureobasidium, Saccharomyces, Fusarium, Cryptococcus, Penicillium, Schizophyllum, Rhodotorula*, and *Gibberella* [32, 35, 40]. It is of note that several of those genera contain species pathogenic for humans and aspergillosis and cryptococcosis can, albeit rarely, cause oral lesions [5]. It has been proposed that there are two ecologically distinct salivary “mycotypes” one where *Candida* (and most commonly *C. albicans*) predominate and the other where *Malassezia* predominate [40]. The *Candida* mycotype showed lower fungal diversity and was associated with cancer and caries, and was also associated with a lower diversity of bacterial communities [40].

Analyzing the mycobiome in parallel with the bacterial microbiome will help investigate synergism and antagonism between species which may consequently promote or prevent oral disease. Indeed, it is well-recognized that there are physical and chemical interactions between fungi and bacteria [41]. Physical adherence interactions between fungi such as *C. albicans* and early bacterial colonizers of oral surfaces such as *Streptococcus gordonii* [42, 43], often mediated by saliva molecules [24], can enable fungi to colonize the mouth. Mutualistic relationships occur between *C. albicans* and a range of mitis group streptococci including *S. gordonii, Streptococcus oralis, Streptococcus mitis*, and *Streptococcus sanguinis* [44, 45]. Not only does *C. albicans* promote formation of bacterial biofilms of mitis *Streptococcus* species on titanium surfaces, but mitis streptococci upregulate expression of the *C. albicans EFG1* and *HWP1* hypha-associated genes and *ALS1* and *ALS3* adhesin genes [44, 45]. *C. albicans* coaggregates with other oral bacteria including *Porphyromonas gingivalis* [46] and *Streptococcus mutans* [47] and by doing so, the metabolic products of the fungus might promote periodontitis and caries, respectively. In polymicrobial biofilms, microbial cross-feeding can occur with the product of one species promoting the growth of another, as demonstrated by increased biofilm growth when *C. albicans* and *S. mutans* are co-cultured [48, 49]. There are also antagonistic interactions between *C. albicans* and oral bacteria. *Lactobacillus* species such as *Lactobacillus plantarum* and *Lactobacillus helveticus* secrete substances that modulate *C. albicans* gene expression and inhibit biofilm formation [50, 51]. This has led researchers to suggest that lactobacilli can be used as probiotics to prevent oral candidiasis [50]. Cross-kingdom chemical signaling often involves quorum sensing molecules such as farnesol from *C. albicans* and autoinducer-2 (AI-2) from oral bacteria [41, 48]. Farnesol inhibits *C. albicans* hyphal formation [52], but AI-2 from *S. gordonii* relieves this repression thus promoting hyphal formation [42]—a virulence factor for the fungus. In contrast, the *S. mutans* quorum sensing molecule competence-stimulating peptide (CSP) inhibits *C. albicans* hypha formation [53]. Thus, chemical communication between fungi and bacteria can potentially promote or inhibit pathogenesis.

The prominence of oropharyngeal candidiasis in HIV/AIDS patients in the 1980s indicated the central role of the host immune system in preventing oral fungal infections. This is also evidenced by increased oral candidiasis in the very young, the elderly, and other individuals who are immunocompromised. There has been considerable research into the host's immune response to fungi such as *C. albicans* which has revealed complex interactions that vary according to the body site [11]. Components of both the innate and adaptive immune response help prevent oral fungal infections. Innate defenses in the oral cavity include saliva production which both flushes microorganisms from the oral cavity and delivers antifungal compounds such as histatins and antibodies, including secretory IgA. Evidence for the importance of saliva production comes from the increased prevalence of oral candidiasis in people with salivary gland hypofunction, or dry mouth (xerostomia) [54]. Another important component of the innate immune response is the recognition, and response, by oral epithelial cells to fungi [11]. Epithelial cells use pattern recognition receptors (PRRs) such as EphA2 to recognize fungal β-glucans [55]. EphA2 activates MAPK and STAT3 pathways and induces epithelial cells to secrete inflammatory cytokines and antimicrobial peptides (AMPs). The *C. albicans* peptide candidalysin can activate the epidermal growth factor receptor of epithelial cells [56] and damage the cells. This damage triggers the release of the alarmin interleukin-1α (IL-1α) which induces a neutrophil response to *C. albicans* via pro-inflammatory cytokine signaling [57]. The cytokines and chemokines IL-1β, IL-6, IL-8, granulocyte colony-stimulating factor (G-CSF), tumor necrosis factor (TNF), and IL-36 are subsequently produced by epithelial cells [58, 59]. IL-8 is a key driver of neutrophil chemotaxis and activation, resulting in their attraction to the site of infection. Although fungal infection generates an inflammatory response, fungi can regulate this response by inducing a subset of neutrophilic myeloid-derived suppressor cells (MDSCs) [60] that functionally suppress T- and NK-cell responses, in a species-specific fashion [61].

There is also an adaptive response to fungal pathogens which is important for generating antibodies and long-term protection [11]. The adaptive response involves antibody production by B-cells and support for mucosal innate responses through T-helper (Th) cells. Th1 and Th17 cells promote phagocytosis of fungal cells through the release of the inflammatory cytokines interferon-γ (INF-γ) and IL-17A/F, respectively [11]. The protective role of IL-17 is evidenced by uncontrolled fungal growth on mucosal surfaces in individuals with defects in Th17 cells and IL-17 signaling [62]. The Th17 cells produce IL-17A, IL-17F, and IL-22 which primarily act on epithelial cells and control the expression of genes involved in antimicrobial defense and tissue repair [63]. Although a major source of IL-17 is conventional Th17 cells, multiple innate lymphocyte subsets produce the cytokine during the early stages of infection [64]. In oropharyngeal candidiasis, for example, innate oral-resident γδ T-cells help control the infection [65]. There is also a neutrophil response to *C. albicans* in the oral mucosa which is coordinated by IL-1 [66]. It is evident that there are multiple immune components involved in protection of the oral cavity from fungal disease and that by monitoring PRR, immune cells, and cytokine expression it may be possible to predict susceptibility to infection.

The widespread use of azoles to treat AIDS patients with oropharyngeal candidiasis led to treatment failure due to *C. albicans* strains developing azole resistance, often due to the expression of efflux pumps [67, 68]. The use of azoles has also selected for increased prevalence of less susceptible *Candida* species such as *Candida glabrata* and *Candida parapsilosis* [69]. Recently, *Candida auris* has emerged as a multidrug-resistant fungal pathogen responsible for hospital outbreaks in many countries [70]. These trends highlight the increasing problem of fungal drug resistance and the limited therapeutic options to combat fungal infections.

## Oral Fungal Infections—Future: Immunosuppression, Viruses and Artificial Intelligence

The number of people who are immunocompromised is increasing and this is due to a range of factors. Notably, individuals are living longer and the ability to mount effective immune responses decreases with age. Also, the incidence of cancer is increasing; the global cancer burden is expected to be 28.4 million cases in 2040, a 47% rise from 2020 [71]. Cancer treatment frequently results in immunosuppression and consequently this increases the risk of fungal infections. Other medical conditions and their treatments can also result in immune suppression. For example, the use of immune suppressing glucocorticoids such as dexamethasone for the treatment of COVID-19 has led to increased incidence of mucormycosis, especially in India [72, 73]. Mucormycosis most commonly affects the nose, sinuses, eyes, and brain. A systematic review of post-COVID-19 fungal infections of the maxillofacial region has revealed that in addition to a large number of cases of candidiasis, there have been many reports of mucormycosis and aspergillosis [74]. This highlights the potential for increased incidence of a different spectrum of oral fungal infections if humans are subjected to different types of immune suppression.

The SARS-CoV-2 pandemic has highlighted several important issues of relevance to oral fungal infections: the oral cavity is continuous with the respiratory tract (nasal cavity and lungs), respiratory viruses pass through the oral cavity, and they are part of the oral microbiome. Thus, the interaction of viruses with other members of the oral microbiome, and the consequences for oral fungal disease need further investigation. While viruses are widely known to infect bacterial and mammalian cells, it is less well-known that viruses can infect fungi. Mycoviral nucleic acid can be detected in *Aspergillus* [75] and *Fusarium* [76] isolates—although to date attention has focussed on *Fusarium* species that infect plants. Analogous to the use of bacteriophages to treat bacterial infections, it has been proposed that mycoviruses could be used to treat fungal infections such as aspergillosis [77]. It is important to note that for this to be effective the virus must cause a lytic infection, and while some mycoviruses demonstrate a hypovirulence or killer phenotype, some induce hypervirulence which must be avoided [77]. It is also important to note that mammalian viruses such as SARS-CoV-2 [78], herpes viruses, cytomegalovirus, and Epstein-Barr virus are present in the oral cavity [79]. These viruses will affect the host immune system. SARS-CoV-2, for example, dysregulates the type I interferon response and increases expression of anti-inflammatory cytokine IL-1RA [80], which may make individuals more susceptible to fungal infections [81, 82].

**Figure 1:**
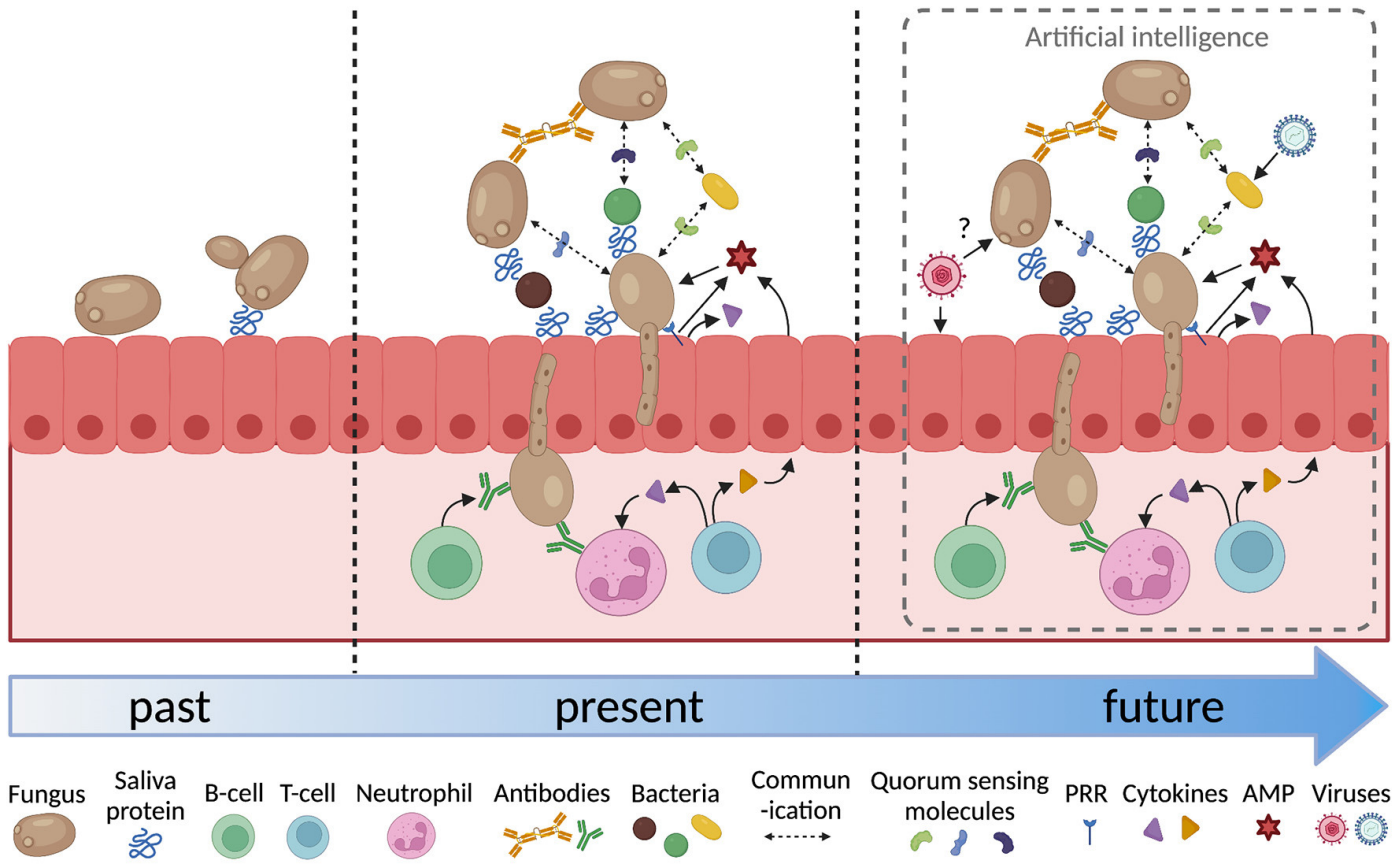
Increasing complexity in the analysis of oral fungal/microbial/host interactions. PRR, pattern recognition receptor; AMP, antimicrobial peptide. Figure created with BioRender.com.

The analysis of host-microbial interactions over the last forty years has become increasingly sophisticated (Figure 1). It has progressed form investigating the attributes of individual microorganisms in isolation to look at the interactions between multiple species and the effect of host factors such as saliva, host tissues, immune cells and signaling molecules. Inter-microbial interactions have focussed on the physical and chemical interactions between fungi and bacteria. These need to be extended to interactions with viruses, as indicated above, but also to include archaea [83], and protozoa [84]. The host response to fungi adds another layer of complexity. The adaptive response involves B-cells, T-cells and neutrophils and cytokine signaling between these cells and the epithelium. Analysis of how each of these factors affects fungal colonization and pathogenesis generates a large amount of complex data that can be difficult to interpret.

Artificial intelligence (AI) is the use of machines to interrogate data and take actions that maximize the chance of achieving a particular objective. When applied to dentistry, AI can use machines to analyse data concerning the oral environment and diagnose a disease. AI can involve machine learning; employing complex algorithms to build a model based on sample data, in order to make predictions or diagnosis or treatment decisions without being explicitly programmed to do so [85]. A literature review of machine learning-based diagnosis and prognosis in clinical dentistry found reports of the use of machine learning algorithms in orthodontics, periodontics, oral medicine and maxillofacial surgery, forensic dentistry, endodontics and cariology [86]. Machine learning has been applied to bacterial microbiome data combined with demographic-environmental factors and fungal information to enable caries prediction in mother-child dyads [87]. The approach has also been used to integrate microbiome data with immune profiling to stratify peri-implantitis patients according to clinical outcomes [88]. There are exciting future prospects of incorporating a wider range of datasets in AI approaches to improve the diagnosis of, and predict risk from, oral fungal infections.

## Conclusions

The analysis of host-microbe interactions has advanced markedly in the last forty years, but key questions remain concerning the ability to detect, diagnose, predict, prevent and treat oral fungal infections. In microbiome analyses, when is a microorganism transient and when has it established a stable population? How reliably is DNA extracted from the range of microorganisms in the oral cavity? Are all the relevant microorganisms being detected? How do co-infections affect the host immune response and disease progression? How can the array of different data obtained from the oral cavity be usefully analyzed? Can aspects of microbial/host interactions be exploited to develop novel therapeutic interventions?

It is evident that there are gaps in our knowledge, and exciting areas for future study. It is important to expand microbiome/mycobiome studies to include archaea, viruses, and protozoa. Nasal and respiratory microbiomes should be included in the analyses. An under-investigated topic is how viruses and fungi interact—this area of research will need to use relevant *in vitro* oral models. With increasing incidence of microbial diseases, such as COVID-19, it is important to investigate how fungal/bacterial and fungal/viral co-infections affect the host immune response and the effect this has on pathogenesis. A particularly profitable area of research would be to combine microbiome and immunological profiles using AI to diagnose and predict oral fungal disease. Finally, we need to use the increased knowledge of interactions between microorganisms and the host to devise new ways to combat oral fungal infections in order to overcome clinical problems caused by the paucity of antifungal agents and increasing antifungal resistance.

## Author Contributions

The author confirms being the sole contributor of this work and has approved it for publication.

## Funding

RC acknowledges funding from the New Zealand Dental Research Foundation (RF8.11).

## Conflict of Interest

The author declares that the research was conducted in the absence of any commercial or financial relationships that could be construed as a potential conflict of interest.

## Publisher's Note

All claims expressed in this article are solely those of the authors and do not necessarily represent those of their affiliated organizations, or those of the publisher, the editors and the reviewers. Any product that may be evaluated in this article, or claim that may be made by its manufacturer, is not guaranteed or endorsed by the publisher.

## References

[1] Baker JL Bor B Agnello M Shi W He X. Ecology of the oral microbiome: beyond bacteria. Trends Microbiol. (2017) 25:362–74. 10.1016/j.tim.2016.12.012 28089325 PMC5687246

[2] Dewhirst FE Chen T Izard J Paster BJ Tanner AC Yu WH. The human oral microbiome. J Bacteriol. (2010) 192:5002–17. 10.1128/JB.00542-10 20656903 PMC2944498

[3] Wade WG. The oral microbiome in health and disease. Pharmacol Res. (2013) 69:137–43. 10.1016/j.phrs.2012.11.006 23201354

[4] Hillman ET Lu H Yao T Nakatsu CH. Microbial ecology along the gastrointestinal tract. Microbes Environ. (2017) 32:300–13. 10.1264/jsme2.ME17017 29129876 PMC5745014

[5] Telles DR Karki N Marshall MW. Oral fungal infections: diagnosis and management. Dent Clin North Am. (2017) 61:319–49. 10.1016/j.cden.2016.12.004 28317569

[6] Krishnan K Chen T Paster BJ. A practical guide to the oral microbiome and its relation to health and disease. Oral Dis. (2017) 23:276–86. 10.1111/odi.12509 27219464 PMC5122475

[7] Naglik JR Challacombe SJ Hube B. *Candida albicans* secreted aspartyl proteinases in virulence and pathogenesis. Microbiol Mol Biol Rev. (2003) 67:400–28. 10.1128/MMBR.67.3.400-428.2003 12966142 PMC193873

[8] Schaller M Borelli C Korting HC Hube B. Hydrolytic enzymes as virulence factors of *Candida albicans*. Mycoses. (2005) 48:365–77. 10.1111/j.1439-0507.2005.01165.x 16262871

[9] Moyes DL Wilson D Richardson JP Mogavero S Tang SX Wernecke J. Candidalysin is a fungal peptide toxin critical for mucosal infection. Nature. (2016) 532:64–8. 10.1038/nature17625 27027296 PMC4851236

[10] Hebecker B Naglik JR Hube B Jacobsen ID. Pathogenicity mechanisms and host response during oral *Candida albicans* infections. Expert Rev Anti Infect Ther. (2014) 12:867–79. 10.1586/14787210.2014.916210 24803204

[11] d'Enfert C Kaune AK Alaban LR Chakraborty S Cole N Delavy M. The impact of the fungus-host-microbiota interplay upon *Candida albicans* infections: current knowledge and new perspectives. FEMS Microbiol Rev. (2021) 45:fuaa060. 10.1093/femsre/fuaa060 33232448 PMC8100220

[12] Odds FC. Candida and Candidosis. Baltimore: University Park Press (1979). p. 1–2.

[13] Holmberg K Meyer RD. Fungal infections in patients with AIDS and AIDS-related complex. Scand J Infect Dis. (1986) 18:179–92. 10.3109/00365548609032326 3526530

[14] Samaranayake LP Holmstrup P. Oral candidiasis and human immunodeficiency virus infection. J Oral Pathol Med. (1989) 18:554–64. 10.1111/j.1600-0714.1989.tb01552.x 2695620

[15] Cannon RD Holmes AR Firth NA. Fungi and fungal infections of the oral cavity. In: Lamont RJ Hajishengallis GN Koo H Jenkinson HF editors. Oral Microbiology and Immunology. 3rd Edn. Washington, DC: ASM Press (2014). p. 397–415.

[16] Odds FC Bernaerts R. CHROMagar *Candida*, a new differential isolation medium for presumptive identification of clinically important *Candida* species. J Clin Microbiol. (1994) 32:1923–9. 10.1128/jcm.32.8.1923-1929.1994 7989544 PMC263904

[17] Hoggard M Vesty A Wong G Montgomery JM Fourie C Douglas RG. Characterizing the human mycobiota: a comparison of small subunit rRNA, ITS1, ITS2, and large subunit rRNA genomic targets. Front Microbiol. (2018) 9:2208. 10.3389/fmicb.2018.02208 30283425 PMC6157398

[18] Marklein G Josten M Klanke U Muller E Horre R Maier T. Matrix-assisted laser desorption ionization-time of flight mass spectrometry for fast and reliable identification of clinical yeast isolates. J Clin Microbiol. (2009) 47:2912–7. 10.1128/JCM.00389-09 19571014 PMC2738125

[19] Cannon RD Holmes AR Mason AB Monk BC. Oral *Candida*: clearance, colonization, or candidiasis? J Dent Res. (1995) 74:1152–61. 10.1177/00220345950740050301 7790592

[20] Sandin RL Rogers AL Patterson RJ Beneke ES. Evidence for mannose-mediated adherence of *Candida albicans* to human buccal cells *in vitro*. Infect Immun. (1982) 35:79–85. 10.1128/iai.35.1.79-85.1982 7033143 PMC350998

[21] Samaranayake LP MacFarlane TW. An *in-vitro* study of the adherence of *Candida albicans* to acrylic surfaces. Arch Oral Biol. (1980) 25:603–9. 10.1016/0003-9969(80)90075-8 7023437

[22] van der Wielen PA Holmes AR Cannon RD. Secretory component mediates *Candida albicans* binding to epithelial cells. Oral Dis. (2016) 22:69–74. 10.1111/odi.12397 26577981

[23] Cannon RD Nand AK Jenkinson HF. Adherence of *Candida albicans* to human salivary components adsorbed to hydroxylapatite. Microbiology. (1995) 141:213–9. 10.1099/00221287-141-1-213 7894715

[24] O'Sullivan JM Jenkinson HF Cannon RD. Adhesion of *Candida albican*s to oral streptococci is promoted by selective adsorption of salivary proteins to the streptococcal cell surface. Microbiology. (2000) 146:41–8. 10.1099/00221287-146-1-41 10658650

[25] Singh DK Nemeth T Papp A Toth R Lukacsi S Heidingsfeld O. Functional characterization of secreted aspartyl proteases in *Candida parapsilosis*. mSphere. (2019) 4:e00484. 10.1128/mSphere.00484-19 31434748 PMC6706470

[26] Ibrahim AS Mirbod F Filler SG Banno Y Cole GT Kitajima Y. Evidence implicating phospholipase as a virulence factor of *Candida albicans*. Infect Immun. (1995) 63:1993–8. 10.1128/iai.63.5.1993-1998.1995 7729913 PMC173255

[27] Mitchell AP. Dimorphism and virulence in *Candida albicans*. Curr Opin Microbiol. (1998) 1:687–92. 10.1016/S1369-5274(98)80116-1 10066539

[28] Socransky SS Haffajee AD Cugini MA Smith C Kent RL Jr. Microbial complexes in subgingival plaque. J Clin Periodontol. (1998) 25:134–44. 10.1111/j.1600-051X.1998.tb02419.x 9495612

[29] Filoche SK Soma KJ Sissons CH. Caries-related plaque microcosm biofilms developed in microplates. Oral Microbiol Immunol. (2007) 22:73–9. 10.1111/j.1399-302X.2007.00323.x 17311629

[30] Vieira Colombo AP Magalhaes CB Hartenbach FA Martins do Souto R Maciel da Silva-Boghossian C. Periodontal-disease-associated biofilm: a reservoir for pathogens of medical importance. Microb Pathog. (2016) 94:27–34. 10.1016/j.micpath.2015.09.009 26416306

[31] Lazarevic V Whiteson K Huse S Hernandez D Farinelli L Osteras M. Metagenomic study of the oral microbiota by Illumina high-throughput sequencing. J Microbiol Methods. (2009) 79:266–71. 10.1016/j.mimet.2009.09.012 19796657 PMC3568755

[32] Ghannoum MA Jurevic RJ Mukherjee PK Cui F Sikaroodi M Naqvi A. Characterization of the oral fungal microbiome (mycobiome) in healthy individuals. PLoS Pathog. (2010) 6:e1000713. 10.1371/journal.ppat.1000713 20072605 PMC2795202

[33] Bandara HMHN Panduwawala CP Samaranayake LP. Biodiversity of the human oral mycobiome in health and disease. Oral Dis. (2019) 25:363–71. 10.1111/odi.12899 29786923

[34] Fechney JM Browne GV Prabhu N Irinyi L Meyer W Hughes T. Preliminary study of the oral mycobiome of children with and without dental caries. J Oral Microbiol. (2019) 11:1536182. 10.1080/20002297.2018.1536182 30598729 PMC6225480

[35] Peters BA Wu J Hayes RB Ahn J. The oral fungal mycobiome: characteristics and relation to periodontitis in a pilot study. BMC Microbiol. (2017) 17:157. 10.1186/s12866-017-1064-9 28701186 PMC5508751

[36] Choo KH Lee HJ Knight NJ Holmes AR Cannon RD. Multilocus sequence typing (MLST) analysis of *Candida albicans* isolates colonizing acrylic dentures before and after denture replacement. Med Mycol. (2017) 55:673–9. 10.1093/mmy/myw128 27915298

[37] Thiyahuddin NM Lamping E Rich AM Cannon RD. Yeast species in the oral cavities of older people: a comparison between people living in their own homes and those in rest homes. J Fungi. (2019) 5:E30. 10.3390/jof5020030 31013697 PMC6617379

[38] Diaz PI Dongari-Bagtzoglou A. Critically appraising the significance of the oral mycobiome. J Dent Res. (2021) 100:133–40. 10.1177/0022034520956975 32924741 PMC8173349

[39] Santus W Devlin JR Behnsen J. Crossing kingdoms: How the mycobiota and fungal-bacterial interactions impact host health and disease. Infect Immun. (2021) 89:e00648–20. 10.1128/IAI.00648-20 33526565 PMC8090948

[40] Hong BY Hoare A Cardenas A Dupuy AK Choquette L Salner AL. The salivary mycobiome contains 2 ecologically distinct mycotypes. J Dent Res. (2020) 99:730–8. 10.1177/0022034520915879 32315566 PMC7243416

[41] Diaz PI Valm AM. Microbial interactions in oral communities mediate emergent biofilm properties. J Dent Res. (2020) 99:18–25. 10.1177/0022034519880157 31590609 PMC6927214

[42] Bamford CV d'Mello A Nobbs AH Dutton LC Vickerman MM Jenkinson HF. *Streptococcus gordonii* modulates *Candida albicans* biofilm formation through intergeneric communication. Infect Immun. (2009) 77:3696–704. 10.1128/IAI.00438-09 19528215 PMC2737996

[43] Holmes AR McNab R Jenkinson HF. *Candida albicans* binding to the oral bacterium *Streptococcus gordonii* involves multiple adhesin-receptor interactions. Infect Immun. (1996) 64:4680–5. 10.1128/iai.64.11.4680-4685.1996 8890225 PMC174431

[44] do Rosario Palma AL Domingues N de Barros PP Brito GNB Jorge AOC. Influence of *Streptococcus mitis* and *Streptococcus sanguinis* on virulence of *Candida albicans: in vitro* and *in vivo* studies. Folia Microbiol. (2019) 64:215–22. 10.1007/s12223-018-0645-9 30232727

[45] Souza JGS Bertolini M Thompson A Barao VAR Dongari-Bagtzoglou A. Biofilm interactions of *Candida albicans* and mitis group streptococci in a titanium-mucosal interface model. Appl Environ Microbiol. (2020) 86:e02950–19. 10.1128/AEM.02950-19 32111586 PMC7170471

[46] Sztukowska MN Dutton LC Delaney C Ramsdale M Ramage G Jenkinson HF. Community development between *Porphyromonas gingivalis* and *Candida albicans* mediated by InlJ and Als3. mBio. (2018) 9:e00202. 10.1128/mBio.00202-18 29691333 PMC5915736

[47] Hwang G Liu Y Kim D Li Y Krysan DJ Koo H. Candida albicans mannans mediate *Streptococcus mutans* exoenzyme GtfB binding to modulate cross-kingdom biofilm development *in vivo. PLoS Pathog*. (2017) 13:e1006407. 10.1371/journal.ppat.1006407 28617874 PMC5472321

[48] Miller DP Fitzsimonds ZR Lamont RJ. Metabolic signaling and spatial interactions in the oral polymicrobial community. J Dent Res. (2019) 98:1308–14. 10.1177/0022034519866440 31356756 PMC6806133

[49] Sztajer H Szafranski SP Tomasch J Reck M Nimtz M Rohde M. Cross-feeding and interkingdom communication in dual-species biofilms of *Streptococcus mutans* and *Candida albicans*. ISME J. (2014) 8:2256–71. 10.1038/ismej.2014.73 24824668 PMC4992082

[50] James KM MacDonald KW Chanyi RM Cadieux PA Burton JP. Inhibition of *Candida albicans* biofilm formation and modulation of gene expression by probiotic cells and supernatant. J Med Microbiol. (2016) 65:328–36. 10.1099/jmm.0.000226 26847045

[51] Srivastava N Ellepola K Venkiteswaran N Chai LYA Ohshima T Seneviratne CJ. *Lactobacillus plantarum* 108 inhibits *Streptococcus mutans* and *Candida albicans* mixed-species biofilm formation. Antibiotics. (2020) 9:478. 10.3390/antibiotics9080478 32759754 PMC7459986

[52] Singkum P Muangkaew W Suwanmanee S Pumeesat P Wongsuk T Luplertlop N. Suppression of the pathogenicity of *Candida albicans* by the quorum-sensing molecules farnesol and tryptophol. J Gen Appl Microbiol. (2020) 65:277–83. 10.2323/jgam.2018.12.002 31217414

[53] Jarosz LM Deng DM van der Mei HC Crielaard W Krom BP. *Streptococcus mutans* competence-stimulating peptide inhibits *Candida albicans* hypha formation. Eukaryot Cell. (2009) 8:1658–64. 10.1128/EC.00070-09 19717744 PMC2772401

[54] Jensen JL Barkvoll P. Clinical implications of the dry mouth. Oral mucosal diseases. Ann N Y Acad Sci. (1998) 842:156–62. 10.1111/j.1749-6632.1998.tb09643.x 9599305

[55] Swidergall M Solis NV Lionakis MS Filler SG. EphA2 is an epithelial cell pattern recognition receptor for fungal b-glucans. Nat Microbiol. (2018) 3:53–61. 10.1038/s41564-017-0059-5 29133884 PMC5736406

[56] Ho J Yang X Nikou SA Kichik N Donkin A Ponde NO. Candidalysin activates innate epithelial immune responses via epidermal growth factor receptor. Nat Commun. (2019) 10:2297. 10.1038/s41467-019-09915-2 31127085 PMC6534540

[57] Dongari-Bagtzoglou A Kashleva H Villar CC. Bioactive interleukin-1alpha is cytolytically released from *Candida albicans*-infected oral epithelial cells. Med Mycol. (2004) 42:531–41. 10.1080/1369378042000193194 15682642

[58] Verma AH Zafar H Ponde NO Hepworth OW Sihra D Aggor FEY. IL-36 and IL-1/IL-17 drive immunity to oral candidiasis via parallel mechanisms. J Immunol. (2018) 201:627–34. 10.4049/jimmunol.1800515 29891557 PMC6039262

[59] Villar CC Kashleva H Mitchell AP Dongari-Bagtzoglou A. Invasive phenotype of *Candida albicans* affects the host proinflammatory response to infection. Infect Immun. (2005) 73:4588–95. 10.1128/IAI.73.8.4588-4595.2005 16040970 PMC1201248

[60] Rieber N Singh A Oz H Carevic M Bouzani M Amich J. Pathogenic fungi regulate immunity by inducing neutrophilic myeloid-derived suppressor cells. Cell Host Microbe. (2015) 17:507–14. 10.1016/j.chom.2015.02.007 25771792 PMC4400268

[61] Singh A Lelis F Braig S Schafer I Hartl D Rieber N. Differential regulation of myeloid-derived suppressor cells by *Candida* species. Front Microbiol. (2016) 7:1624. 10.3389/fmicb.2016.01624 27790210 PMC5061774

[62] Puel A Cypowyj S Bustamante J Wright JF Liu L Lim HK. Chronic mucocutaneous candidiasis in humans with inborn errors of interleukin-17 immunity. Science. (2011) 332:65–8. 10.1126/science.1200439 21350122 PMC3070042

[63] Conti HR Shen F Nayyar N Stocum E Sun JN Lindemann MJ. Th17 cells and IL-17 receptor signaling are essential for mucosal host defense against oral candidiasis. J Exp Med. (2009) 206:299–311. 10.1084/jem.20081463 19204111 PMC2646568

[64] Verma A Gaffen SL Swidergall M. Innate immunity to mucosal *Candida* infections. J Fungi. (2017) 3:60. 10.3390/jof3040060 29371576 PMC5753162

[65] Conti HR Peterson AC Brane L Huppler AR Hernandez-Santos N Whibley N. Oral-resident natural Th17 cells and gamma delta T cells control opportunistic *Candida albicans* infections. J Exp Med. (2014) 211:2075–84. 10.1084/jem.20130877 25200028 PMC4172215

[66] Altmeier S Toska A Sparber F Teijeira A Halin C LeibundGut-Landmann S. IL-1 Coordinates the neutrophil response to *C. albicans* in the oral mucosa. PLoS Pathog. (2016) 12:e1005882. 10.1371/journal.ppat.1005882 27632536 PMC5025078

[67] Cannon RD Lamping E Holmes AR Niimi K Baret PV Keniya MV. Efflux-mediated antifungal drug resistance. Clin Microbiol Rev. (2009) 22:291–321. 10.1128/CMR.00051-08 19366916 PMC2668233

[68] Sanglard D Kuchler K Ischer F Pagani JL Monod M Bille J. Mechanisms of resistance to azole antifungal agents in *Candida albicans* isolates from AIDS patients involve specific multidrug transporters. Antimicrob Agents Chemother. (1995) 39:2378–86. 10.1128/AAC.39.11.2378 8585712 PMC162951

[69] Silva S Negri M Henriques M Oliveira R Williams DW Azeredo J. Candida glabrata, Candida parapsilosis and Candida tropicalis biology, epidemiology, pathogenicity and antifungal resistance. FEMS Microbiol Rev. (2012) 36:288–305. 10.1111/j.1574-6976.2011.00278.x 21569057

[70] Forsberg K Woodworth K Walters M Berkow EL Jackson B Chiller T. *Candida auris*: the recent emergence of a multidrug-resistant fungal pathogen. Med Mycol. (2019) 57:1–12. 10.1093/mmy/myy054 30085270

[71] Sung H Ferlay J Siegel RL Laversanne M Soerjomataram I Jemal A. Global cancer statistics 2020: GLOBOCAN estimates of incidence and mortality worldwide for 36 cancers in 185 countries. CA Cancer J Clin. (2021) 71:209–49. 10.3322/caac.21660 33538338

[72] Gambhir RS Aggarwal A Bhardwaj A Kaur A Sohi RK Mehta S. Covid-19 and mucormycosis (Black Fungus): an epidemic within the pandemic. Rocz Panstw Zakl Hig. (2021) 72:239–44. 10.32394/rpzh.2021.0169 34553877

[73] Patel A Agarwal R Rudramurthy SM Shevkani M Xess I Sharma R. Multicenter epidemiologic study of coronavirus disease-associated mucormycosis, India. Emerg Infect Dis. (2021) 27:2349–59. 10.3201/eid2709.210934 34087089 PMC8386807

[74] Jain A Taneja S. Post-COVID fungal infections of maxillofacial region: a systematic review. Oral Maxillofac Surg. (2021) 7:1–7. 10.1007/s10006-021-01010-5 34622312 PMC8497068

[75] Bhatti MF Jamal A Bignell EM Petrou MA Coutts RH. Incidence of dsRNA mycoviruses in a collection of *Aspergillus fumigatus* isolates. Mycopathologia. (2012) 174:323–6. 10.1007/s11046-012-9556-5 22610906

[76] Sharma M Guleria S Singh K Chauhan A Kulshrestha S. Mycovirus associated hypovirulence, a potential method for biological control of *Fusarium* species. Virusdisease. (2018) 29:134–40. 10.1007/s13337-018-0438-4 29911145 PMC6003058

[77] van de Sande WWJ Vonk AG. Mycovirus therapy for invasive pulmonary aspergillosis? Med Mycol. (2019) 57(Supplement_2):S179–88. 10.1093/mmy/myy073 30816971

[78] Takeuchi Y Furuchi M Kamimoto A Honda K Matsumura H Kobayashi R. Saliva-based PCR tests for SARS-CoV-2 detection. J Oral Sci. (2020) 62:350–1. 10.2334/josnusd.20-0267 32581183

[79] Slots J. Periodontal herpesviruses: prevalence, pathogenicity, systemic risk. Periodontol 2000. (2015) 69:28–45. 10.1111/prd.12085 26252400

[80] Olbei M Hautefort I Modos D Treveil A Poletti M Gul L. SARS-CoV-2 causes a different cytokine response compared to other cytokine storm-causing respiratory viruses in severely ill patients. Front Immunol. (2021) 12:629193. 10.3389/fimmu.2021.629193 33732251 PMC7956943

[81] Baddley JW Thompson GR III Chen SC White PL Johnson MD Nguyen MH. Coronavirus disease 2019-associated invasive fungal infection. Open Forum Infect Dis. (2021) 8:ofab510. 10.1093/ofid/ofab510 34877364 PMC8643686

[82] Peman J Ruiz-Gaitan A Garcia-Vidal C Salavert M Ramirez P Puchades F. Fungal co-infection in COVID-19 patients: should we be concerned? Rev Iberoam Micol. (2020) 37:41–6. 10.1016/j.riam.2020.07.001 33041191 PMC7489924

[83] Diaz PI. Subgingival fungi, Archaea, and viruses under the omics loupe. Periodontol 2000. (2021) 85:82–9. 10.1111/prd.12352 33226731

[84] Vozza I Zino G Puddu P Quaranta M. Study on the frequency of protozoa and mycetes in the oral cavity. Minerva Stomatol. (2005) 54:575–81. 16224377

[85] Seneviratne CJ Balan P Suriyanarayanan T Lakshmanan M Lee DY Rho M. Oral microbiome-systemic link studies: perspectives on current limitations and future artificial intelligence-based approaches. Crit Rev Microbiol. (2020) 46:288–99. 10.1080/1040841X.2020.1766414 32434436

[86] Reyes LT Knorst JK Ortiz FR Ardenghi TM. Scope and challenges of machine learning-based diagnosis and prognosis in clinical dentistry: a literature review. J Clin Transl Res. (2021) 7:523–39. 10.18053/jctres.07.202104.012 34541366 PMC8445629

[87] Wu TT Xiao J Sohn MB Fiscella KA Gilbert C Grier A. Machine learning approach identified multi-platform factors for caries prediction in child-mother dyads. Front Cell Infect Microbiol. (2021) 11:727630. 10.3389/fcimb.2021.727630 34490147 PMC8417465

[88] Wang CW Hao Y Di Gianfilippo R Sugai J Li J Gong W. Machine learning-assisted immune profiling stratifies peri-implantitis patients with unique microbial colonization and clinical outcomes. Theranostics. (2021) 11:6703–16. 10.7150/thno.57775 34093848 PMC8171076

